# Neuromorphic Architecture Accelerated Automated Seizure Detection in Multi-Channel Scalp EEG

**DOI:** 10.3390/s22051852

**Published:** 2022-02-26

**Authors:** Ravi Ambati, Shanker Raja, Majed Al-Hameed, Titus John, Youness Arjoune, Raj Shekhar

**Affiliations:** 1 Sheikh Zayed Institute for Pediatric Surgical Innovation, Children’s National Hospital, Washington, DC 20010, USA; rambati@childrensnational.org (R.A.); tjohn2@childrensnational.org (T.J.); yarjoune@childrensnational.org (Y.A.); 2 National Neuroscience Institute, King Fahad Medical City, Riyadh 12231, Saudi Arabia; shanker.raja01@gmail.com (S.R.); malhameed@kfmc.med.sa (M.A.-H.)

**Keywords:** seizure detection, electroencephalogram, anomaly detection, neuromorphic computing, machine learning, sample entropy, correlation dimension, discrete wavelet-decomposition

## Abstract

Epileptic focal seizures can be localized in the brain using tracer injections during or immediately after the incidence of a seizure. A real-time automated seizure detection system with minimal latency can help time the injection properly to find the seizure origin accurately. Reliable real-time seizure detection systems have not been clinically reported yet. We developed an anomaly detection-based automated seizure detection system, using scalp-electroencephalogram (EEG) data, which can be trained using a few seizure sessions, and implemented it on commercially available hardware with parallel, neuromorphic architecture—the NeuroStack. We extracted nonlinear, statistical, and discrete wavelet decomposition features, and we developed a graphical user interface and traditional feature selection methods to select the most discriminative features. We investigated Reduced Coulomb Energy (RCE) networks and K-Nearest Neighbors (k-NN) for its several advantages, such as fast learning no local minima problem. We obtained a maximum sensitivity of 91.14%±1.77% and a specificity of 98.77%±0.57% with 5 s epoch duration. The system’s latency was 12 s, which is within most seizure event windows, which last for an average duration of 60 s. Our results showed that the *CD* feature consumes large computation resources and excluding it can reduce the latency to 3.6 s but at the cost of lower performance 80% sensitivity and 97% specificity. We demonstrated that the proposed methodology achieves a high specificity and an acceptable sensitivity within a short delay. Our results indicated also that individual-based RCE are superior to population-based RCE. The proposed RCE networks has been compared to SVM and ANN as a baseline for comparison as they are the most common machine learning seizure detection methods. SVM and ANN-based systems were trained on the same data as RCE and K-NN with features optimized specifically for them. RCE nets are superior to SVM and ANN. The proposed model also achieves comparable performance to the state-of-the-art deep learning techniques while not requiring a sizeable database, which is often expensive to build. These numbers indicate that the system is viable as a trigger mechanism for tracer injection.

## 1. Introduction

Epilepsy is one of the most common neurological disorders, affecting up to one percent of the population worldwide and almost two million people in the United States alone [1]. Up to 30% of epilepsy patients experience medically refractory recurrent seizures [2] that do not respond to anti-seizure medication. In patients presenting with medically intractable seizures, complete surgical resection of the epileptic zone may be curative, offering the best long-term prognosis, with either complete absence of seizures or partial response to surgery with decreased seizure frequency and/or decreased use of anti-epileptic medication.

Presurgical evaluation entails extensive workup, including clinical workup, interictal (between seizures) scalp EEG, ictal (during seizures) video EEG monitoring, and neuropsychological testing; in addition, patients undergo morphologic (MRI, CT) and functional (interictal PET and ictal single photon emission computed tomography (ictal-SPECT)) multimodality imaging [3]. Usually, patients are offered neurosurgical options if the clinical presentation, ictal-interictal EEG, and imaging features are concordant for localization of the seizure focus. Often, despite extensive presurgical workup and imaging, either the data is discordant or inconclusive; in this large subset of patients, ictal-SPECT is often helpful for localizing seizures [3] and phases, which demonstrates areas of acute ictal hyperperfusion (enhanced perfusion during seizures). Ictal-SPECT imaging is instrumental in identifying non-lesional intractable seizures and in pediatric patients.

Seizures are known to propagate rapidly to the ipsilateral and contralateral cortex, especially in extratemporal foci compared to temporal foci. This propagation is very rapid and often diffuse. Since blood flow follows electrical activity [4], it is imperative to inject the perfusion tracers as soon as the onset of seizures on EEG and/or video monitoring is observed. Hence, to obtain an accurate ictal-SPECT scan, the elapsed time from seizure onset to tracer injection is critical and must be as short as possible [5]. The reliability of tracer injection for seizure localization significantly improves the elapsed period from seizure onset to tracer injection; Early radio tracer injection has been considered the most critical factor for seizure localization. Pastor et al. [6] and Setoain et al. [7] reported improved seizure localization using automated tracer injection (average of 33 s; range: 19–63 s; p<0.05) compared to manual injections (average of 41 s; range: 14–103 s; p=0.14) and a successful localization seizure focus in 21 of the 27 patients (78%) by automated technique as opposed to 19 of the 29 patients (65%) by manual technique. Ho et al. [8] have documented the different cerebral perfusion patterns in temporal lobe seizures during ictal and periictal phases. Delayed injections lead to diffuse/multiple-foci of hyper-perfusion on ictal-SPECT, thus invalidating the procedure.

Automated seizure detection on ictal EEG has been attempted for more than four decades. After preprocessing the EEG signal for noise and artifact removal, different techniques have been used for the detection task, including rule-based wavelet and spectral analysis, artificial neural networks (ANN), and support vector machines (SVM) [9,10,11] (Table 1). Research in neurostimulation and automated drug delivery systems has further grown this field, and ANN and SVM are emerging as the front runner classifiers in automated systems [12,13,14]. Though the reported detection accuracies of various techniques have been impressive, reaching as high as 90% or more, these results are based on well-defined and cleansed samples and are often obtained off-field in the laboratory [9]. When deployed in a real-world clinical setting, the accuracies can plummet significantly. Currently, neural networks are software emulations and are computationally intensive. So, a finite time is elapsed for processing the input data streams; the temporal delay is well known to exponentially increase with increasing volumes and the complexity of the incoming data streams. It has also been noted in several studies [15,16,17,18,19] that individual-based systems perform better than a generalized system because of the significant inter-individual variance of epileptic signals and their general random nature. When deployed in real-world settings, these systems generally tend to have a minimal amount of patient-specific ictal/seizure EEG data than the interictal/normal data.

The traditional methods for seizure detection such as ANN, need large amounts of training data for acceptable performance. Also, it has been shown that ANN requires 4 fold more computational power than SVM. Wang et al. [20] proposed a random forest with grid search optimization. In addition, most studies reporting the classification results with these machine learning (ML) models use a large database, such as the CHB-MIT scalp EEG Database, for training and reporting the model metrics [10,21]. Typically, if we take a few EEG sessions for training and aim to perform the SPECT injection in the subsequent few sessions, we would have a substantial amount of normal data but very little seizure data. In our clinical recordings, a session contained 4 h of normal data and 71 s of seizure data on average. Many adaptive pattern classifiers have been developed to provide high-performance and real-time responses with real-world data. Much recent emphasis has been placed on deep learning, but numerous other classifiers have been developed. These include decision trees, Boltzmann machines, RCE networks, feature-map, LVQ, high-order networks, radial basis function classifiers, and modified nearest neighbor approaches, to name a few. These classifiers provide trade-offs in memory and computation requirements, training complexity, and ease of implementation and adaptation. K-NN methods allow reduced error rates. For instance, several studies have demonstrated that k-NN, which train rapidly but require large amounts of memory and computation, sometimes perform as well as back-propagation classifiers, which are more complex to train but require less memory. Decision trees, which have small memory and computation requirements, often perform as well as more complex back-propagation classifiers but are more prone to over-fitting. Radial basis function classifiers require intermediate amounts of memory and training time. RCE networks require less memory than k-nearest neighbor classifiers but adapt its structure over time using simple adaptation rules that recruit new nodes to match the complexity of the classifier to that of the training data. It was reported, in [22], that RCE networks adapt faster and require fewer exemplar nodes than the nearest neighbor classifiers as more nodes, if needed, are recruited to generate more complex decision regions, and the size of hyper-spheres formed by existing nodes is modified during adaptation. It has been demonstrated, both theoretically and experimentally, that RCE forms complex decision regions rapidly. They can be trained to solve many problems more than an order of magnitude faster than back-propagation classifiers. RCE networks are currently being applied to many real-world problems for real-time execution, due to their fast learning and the absence of local minima.

Recent advances in machine learning science and deep learning techniques have shown their superiority for learning very robust seizure representation features. For example, artificial neural networks (ANNs) were used to detect seizures after using traditional feature extraction techniques. Some researchers have used semi-supervised deep learning strategies for epileptic EEG classification. The most widely used method involves training a neural network in an unsupervised way using unlabeled data and then training it again in a supervised way using labeled data.

Several deep learning-based systems have been proposed to address the limitation of the classification schemes mentioned above [23,24,25,26]. For instance, Abdelhameed et al. [23] proposed a 2D supervised deep convolutional autoencoder (SDCAE) to detect epileptic seizures in multichannel EEG signals recordings automatically. They showed that deep learning could achieve 98% detection accuracy with high sensitivity. The computational training and testing times of these models were not reported. Although deep learning approaches seem to be attractive, it requires a sizeable database, which is not always available. Furthermore, deep learning requires specific hardware for faster training, yet building large comprehensive datasets is tedious and expensive. Additionally, the large volumes of continuous EEG recordings required for deep learning algorithms are limited and remain a significant limitation. Finally, in order to elucidate the optimal network structure for a deep neural network, substantial labor may be required. To the best of the authors’ knowledge, few to no studies have examined the use of machine learning for automatic seizure detection with experimental implementation on hardware. The choice of hardware implementation over software implementation is because dedicated hardware provides real-time and faster processing compared with general software [27].

We identified k-Nearest Neighbors (k-NN) and Reduced Coulomb Energy (RCE) networks for this task [28]. Wang et al. [11,29] reported high accuracies using k-NN and SVM, respectively. Shoka et al. [30] developed an automatic seizure diagnosis based on channel selection. Shoka et al. tested several machine learning techniques such as SVM, Ensemble decision trees, k-NN, LDA, Logistic Regression, decision trees, and Naive Bayes. These algorithms showed 80% accuracy on unfiltered data. They showed also that filtered data improved the detection by 1% to 2%. Rivero et al. [31] also reported high accuracy using k-NN. The choice of these algorithms was also motivated by the commercial availability of specialized hardware tailored for implementing these algorithms. Based on neuromorphic architecture [32], this hardware has been engineered to improve the accuracy of pattern recognition and, more importantly, decrease the elapsed time between signal input and the output of results, and has been used recently by many researchers [33]. We use NeuroStack [34] board from General Vision (Petaluma, CA, USA) for our application, which has multiple neuromorphic chips and enables multiple such boards to be daisy-chained, significantly increasing its ability for pattern learning. The NeuroStack has an onboard FPGA for digital signal processing operations. The FPGA has parallel architecture and has multiple processing elements, which can be used to implement a high-throughput map-reduce framework to speed up the preprocessing operations on multiple EEG channels.

The major contributions of this study are summarized as follows:

- We developed a clinical dataset that consists of 205 recordings with an average of 7 h and 35 min for normal brain activity and 5 min 11 s for seizure. The 205 EEG recordings has been collected from 45 patients;
- We demonstrated that traditional k-NN and RCN could achieve high seizure identification accuracy with high sensitivity (91.14%) and acceptable specificity (98.77%), achieving comparable performance to support vector machine, ANN, and deep learning. We did not directly compare the proposed technique to deep learning on the same datasets because the hardware used in this study does not support deep learning, but we could obtain results from recent studies and surveys. The results show that machine learning can be used in limited data and computing resources cases, which is often the case. Another advantage of traditional machine learning over deep learning is eliminating longer labeling tasks;
- We investigated several types of features such as nonlinear features (sample entropy and correlation dimension) and first and second-order feature extraction. We also explored several feature selections such as mutual information-based feature selection, Chi-square score-based feature selection, ANOVA F1-Value, and Recursive Feature Elimination. We showed that well-engineered features could help machine learning achieve high accuracy while supporting real-time seizure detection. We showed that a latency as small as 3.6 s can be achieved;
- In comparing the proposed method to other state-of-the-art machine learning, we showed that the proposed methodology is superior to SVM and ANN. They are the most widely used algorithms in seizure detection. Because of the limited training dataset, we only employed a 4-layer neural network. Increasing the depth of the network requires more training data, which we did not have;
- We developed a graphical user interface that can assist epileptologists to apply their expertise in the field and facilitate the labeling jobs as they can spend less time with this task.

The remaining sections of this paper are outlined as follows: Section 2 describes data collection, feature extraction, and feature selection as well as the ML methods used for classification. Section 3 describes the experimental setup, experiments, training, and evaluation metrics. It also describes examples of results as well as their analysis. Section 4 discusses the results in the context of related and state-of-the-art-techniques. Section 5 summarizes the main findings of this study and concludes the paper.

## 2. Materials and Methods

### 2.1. Data Collection

We used archived scalp-EEG data collected over two years at the King Fahad Medical City (KFMC), Riyadh, Saudi Arabia. This study utilized archived clinical data that was approved as an exempt study by the institutional review board (IRB) of KFMC.

Inclusion criteria: all adult patients with suspected focal intractable seizures admitted to the video-EEG monitoring suite for seizure localization.

Exclusion criteria: all pediatric patients with suspected focal intractable seizures.

Recordings: all EEG recordings were made in the video-EEG monitoring suite.

**Figure 1:**
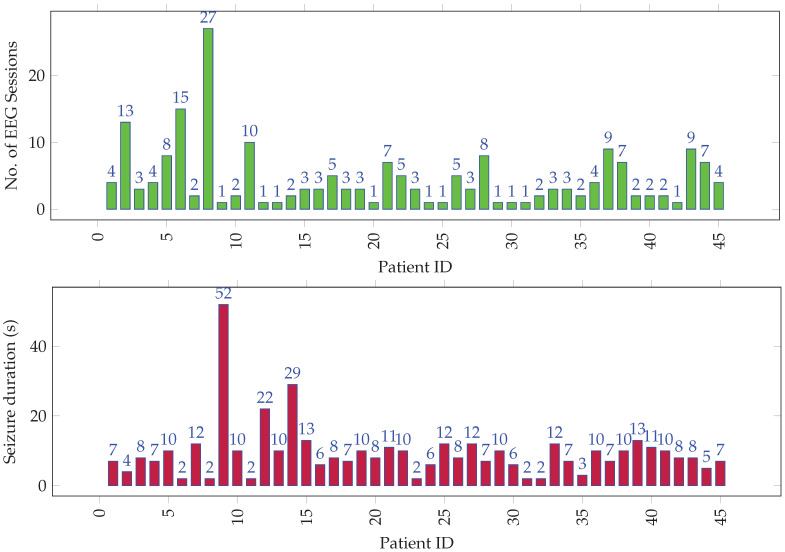
Distribution of (**top**) EEG sessions and (**bottom**) seizure duration (in tens of seconds) per session among all individuals.

The EEG signals had 21 channels per recording, which were captured using a 10–20 electrode system [42] at a sampling rate of 500 Hz. The dataset comprised 205 EEG recordings from 45 unique individuals. The data was annotated by the epileptologists at KFMC. Figure 1 gives a graphical overview of seizure data distribution among all the individuals. Over the 205 cases, the average seizure duration was 1 min 11 s, and the average normal activity (up to the seizure onset) duration was 1 h 35 min. The onset of seizures from the start of EEG recording was short, since some patients had a history of persistent seizures and presented with seizure activity immediately after the recording’s start. Over the 45 subjects who make up the 205 recordings, the average seizure duration was 5 min 11 s, and the median duration was 2 min. The average interictal duration was 7 h and 35 min, and the median duration was 1 h 33 min.

### 2.2. Preprocessing

**Figure 2:**
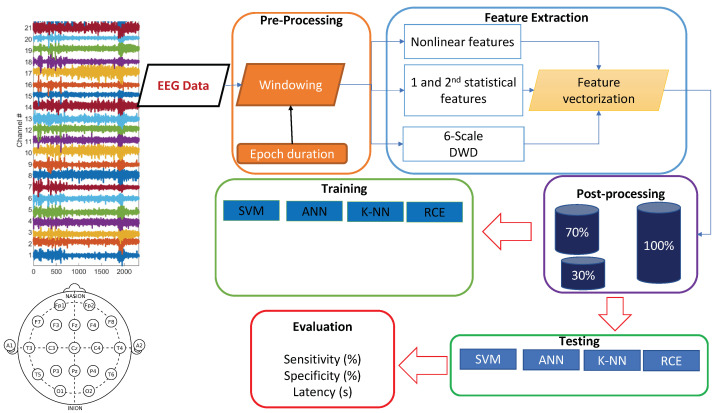
Training and testing pipeline.

EEG signals are often contaminated with artifacts. For instance, eye-blinks and the movement of eyeballs generate electrical signals, which are collectively known as ocular artifacts (OA) [43]. Other artifacts include muscle artifacts, cardiac artifacts, and extrinsic artifacts [44]. A variety of techniques has been proposed in the literature to remove these artifacts, which can be broadly classified mainly into two categories. The first category estimates the artifactual signals using a reference channel, whereas the second decomposes EEG signals into other domains. Techniques of the latter category include regression, blind source separation (BSS), empirical-mode decomposition (EMD), DWD [43,45,46,47], and hybrid methods. A complete review on these methods can be found in [44]. In this paper, EEG signals went through preprocessing before feature extraction. We focused on wavelet decomposition, which has many advantages over other alternatives in that it supports automatic processing, can be performed on a single channel and has versatility to attenuation artifacts [44]). After decomposition of EEG data using wavelet transformation, thresholding was applied to discard the signal that contained artifacts. In this preprocessing, windowing was applied, and the applied window equaled the epoch length (Figure 2). The EEG signals were windowed into fixed-length epochs. The epoch length varied from 1 s to 7 s. The preprocessing removed low-frequency artifacts. The mathematical model of the DWD is described in detail in the following subsections.

#### 2.2.1. Feature Extraction

We used traditional machine learning methods for this real-time application instead of deep learning methods since the traditional methods have simple hardware and computational requirements. Hence, there was a need for low-level feature extraction. The raw data was windowed into fixed-length epochs, and features were extracted from each epoch. Since the raw data had 21 channels, each epoch has 21 channels as well. We extracted nonlinear dynamical features and statistical features from the EEG data.

Nonlinear features: We extracted nonlinear features based on chaos theory, which have been proven to represent brain activity well [1,21,48,49]. These nonlinear features were sample entropy (*SampEn*) and correlation dimension (*CD*).

The sample entropy of a time-series is the negative natural logarithm of the conditional probability that two sequences similar to ‘*m*’ points remain similar at the next point, where self matches are not included in calculating the probability. *SampEn* [50] can be computed as:

(1) $$SampEn\left(m,r,n\right)=-ln\left(\frac{A}{B}\right)$$

where ‘*m*’ is the embedding dimension (length of vectors to compare). We used an embedding dimension of 2, which has been shown to be appropriate for small datasets [51], *r* is the tolerance value, *n* is the original data length, and *A* and *B* are given as,

(2) $$A=\left\{\frac{\left(n-m-1)(n-m\right)}{2}\right\}A^{m}\left(r\right),and\;B=\left\{\frac{\left(n-m-1)(n-m\right)}{\left(2\right)}\right\}B^{m}\left(r\right),$$

where, $A^{m}\left(r\right)$ denotes the probability that the two sequences match for m+1 points, and $B^{m}\left(r\right)$ denotes the probability that the two sequences match for *m* points [50].

Entropy is a concept handling predictability and randomness, with higher values of entropy always related to less system order and more randomness [52]. In the event of a seizure, the EEG signal on certain channels shows more randomness, suggesting a high value of *SampEn* for the seizure epochs compared to epochs with normal brain activity. *SampEn* is calculated using the algorithm proposed by Richman et al. [51].

The *CD* of a set of points measures the space dimensionality occupied by these points. It determines if a seemingly random time-series signal was truly random or generated by a nonlinear dynamical deterministic system. A truly random signal cannot be embedded in a smaller dimension than the embedding dimension, while a signal generated by a nonlinear dynamical system can be embedded within a smaller dimension space. It has been observed that seizure data has smaller *CD* compared to normal data. As a result, one can conclude that any discernible randomness in normal data is likely due to random noise, whereas the randomness in seizure data is due to seizure generating mechanisms in the brain. The *CD* is calculated using the Grassberger-Procaccia algorithm [53].

For a time series given by $ts=(x_{1},x_{2},...,x_{n})$, the *CD* can be computed as [54],

(3) $$CD=\lim_{r\overset{}{\to }0}\frac{log(C_{r})}{log(r)},$$

where $C_{r}$ is given by [54],

(4) $$C_{r}=\lim_{r\overset{}{\to }0}\frac{log(C(r))}{N^{2}},$$

and

(5) $$C\left(r\right)=\sum_{}^{}\Theta (r-||x_{i}-x_{j}||),$$

where Θ denotes the Heaviside function.

Statistical features: Statistical features were extracted from the sub-signals obtained from discrete wavelet decomposition (DWD) of each channel of the EEG signal. DWD has been successfully used for extracting features in multiple studies with EEG data [10,55,56]. DWD provides a high-resolution signal at each analysis scale while not compromising the temporal resolution.

DWD is based on discrete wavelet transform. A wavelet is an oscillating function that is rapidly diminishing. The signal is split into scaled and translated versions $\left(\psi_{a,b}\left(t\right)\right)$ of a single function ψ, termed mother wavelet, in continuous wavelet analysis [57]:

(6) $$\psi_{a,b}\left(t\right)=\frac{1}{\sqrt{a}}\psi \left(\frac{t-b}{a}\right),$$

where *a* and *b* are the scale and translation parameters, respectively. DWD was obtained by discretizing the scale and translation parameters. Several mother wavelet functions can be used [45,46,47] Each epoch was decomposed into seven sub-signals using a six-level DWD. Six-level DWD has been shown to be appropriate for feature representation in time and frequency domain [58,59]. In addition, we conducted wavelet decomposition of EEG with five scales, and selected few frequency bands of them for subsequent processing inspired by the work done by Liu et al. [39]. Table 2 gives the frequency composition of each sub-signal represented by corresponding detail or approximation coefficients.

First and second-order statistical estimates—absolute value of the mean (AM), mean of the absolute values (MA), and root mean square (RMS) value—were computed for each channel of each sub-signal. This resulted in 21 features per epoch per channel. There were 23 total features per epoch per channel—2 nonlinear features and 21 features from the DWD. Feature selection methods were used to select the most important features before training the ML classifiers.

#### 2.2.2. Feature Selection

We developed a simple graphical user interface (GUI) using Python programming language, which helped us visualize the seizure, non-seizure data, and the corresponding features. The initial features were selected such that there was a visible distinction in the features corresponding to normal and seizure data. The feature selection experiments were performed with epoch durations of 3 s, 5 s, and 7 s, which are commonly used sub-sample durations [56,60].

**Figure 3:**
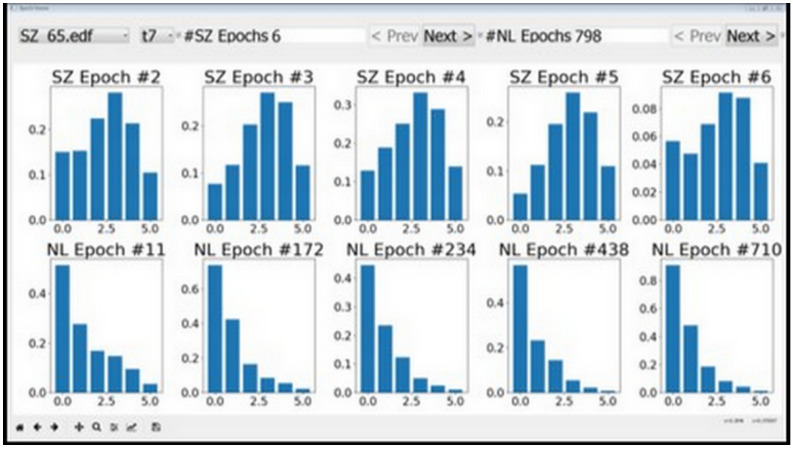
Features as visualized by epoch viewer tool. The plots in the top row correspond to seizures and the bottom row plots correspond to normal data. It can be seen that the second feature does not vary much between seizures and normal data. The behavior persisted across different session and individuals, and therefore the second feature was removed from consideration.

Features with low discriminative power—RMS, MA, and AM corresponding to the [0, 3.90625] Hz band—could be easily identified using the tool. This apparent visual distinction can be attributed to the DC content in this frequency band, which does not appear to change much between seizure and normal activity. We started with 23 features per channel and arrived at 20 using the visualization tool depicted in Figure 3.

The following filter and wrapper feature selection methods were applied on this reduced set, each method resulting in a distinct optimal feature set.

- Mutual information-based feature selection: The features are ranked based on the mutual information between them and the target output. The mutual information is calculated using entropy estimation from k-Nearest Neighbor distances [61,62];
- Chi-squared score-based feature selection: The features are ranked based on the chi-squared value [63] between them and the target output. Since chi-squared statistic measures the dependence between stochastic variables, it helps to remove the non-discriminative features, which are most likely independent of the target class;
- ANOVA F-Value: This method selects the features with the highest one-way analysis of variance (ANOVA) [64,65] score between the target output and each feature;
- Recursive Feature Elimination (RFE): This is a wrapper method where features are recursively eliminated until the performance of a classifier stops improving [66].

#### 2.2.3. Machine Learning

**Figure 4:**
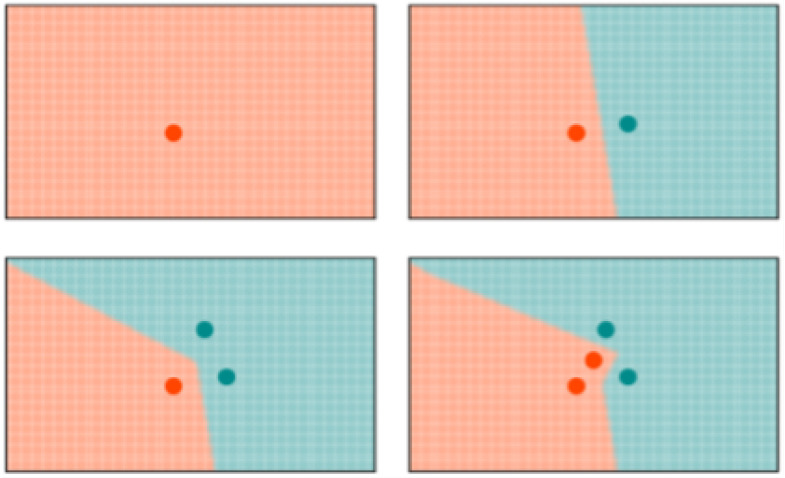
Evolution of the decision space for k-NN network with k=1. All the input examples are saved in memory and the decision space is determined by the k-nearest neighbors.

Since our goal was to develop a real-time seizure detection system, the selected ML algorithms had to be simple in terms of hardware and power requirements [22]. Owing to the preponderance of normal data and disproportionately small amount of seizure data, ML algorithms from the anomaly detection class of algorithms were used. The term anomaly detection is used here because the seizure does not last for a long duration compared with the session recording. For this specific medical application, i.e., seizure focus localization, the true positive rate (rate of correct identification of seizure activity) should be as high as possible with the lowest-possible false positive rate (rate of misclassification of normal activity as seizure activity). We used k-Nearest Neighbors (k-NN) and Reduced Coulomb Energy (RCE) networks for this task. Similar template-based classifiers were used by Qu et al. [16]. We compared the results of classifiers based on these algorithms to the baseline results obtained from the classifiers based on traditional ML algorithms SVM and ANN.

k-NN: K-NN is a non-parametric, memory-based classification algorithm. It commits all the training examples to memory and uses them as templates for classification. When a test example is presented, the algorithm computes the L2 distance to each of the saved templates. The k closest examples are then selected, and the test example is assigned to the class represented by the majority of the *k* examples. The algorithm has one hyperparameter—the number of nearest neighbors, *k*. As seen in Figure 4, a binary k-NN divides the feature space into two distinct regions corresponding to the two classes.

**Figure 5:**
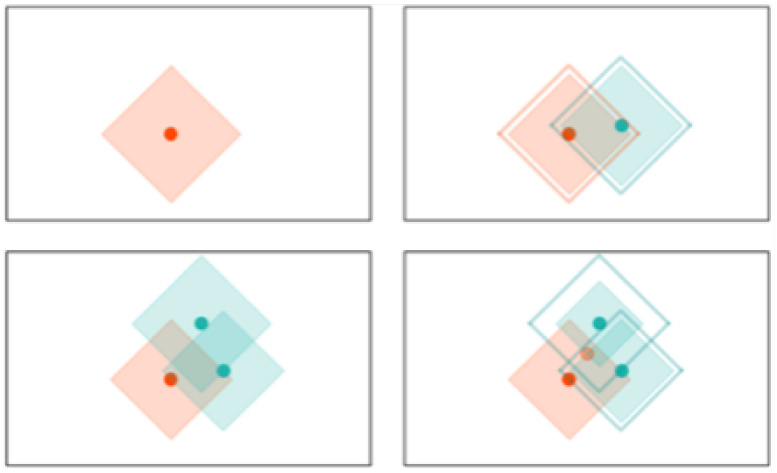
Evolution of the decision space for an RCE network with $L_{1}$ distance measure in 2-D space. Note how the decision space is divide into three regions and how it is modified with the presence of a different class nearby.

RCE: RCE improve the accuracy of K-NN by adding a distance threshold. In this way, RCE addresses one of the main deficiencies of K-NN. As it can be seen in Figure 4, an input example still has neighbors and is attributed to one class even when it is very distant from any saved example. Adding a distance threshold (Figure 5) allows for saving only examples who are close to neighbors and find correct classes for new input examples. Another advantage of RCE is that not all examples are committed to memory. The shape of the IF around a vector is defined by the distance metric used for the network—e. If a new training vector falls in the IF of a vector belonging to the other class, the IF of the existing vector is shrunk so as not to include the new vector and the new vector is assigned an IF so as not to include the other vector in its IF. A binary RCE network, in contrast to a k-NN, can output three classes: class 1, class 2, or Unknown class. When deployed for classification, if the vector falls within the influence field of either or both of the classes, it is classified into the closest class in terms of the network distance metric. This can happen frequently with seizures, owing to their random nature—seizure patterns can vary significantly from one session to the other for the same individual [67].

SVM: SVM is a two-class classifier, which builds a nonlinear boundary between the two classes of interest in a multidimensional feature space [68]. We tested several kernel functions—radial basis function, polynomial, and linear kernels—and selected linear kernel with automatic class weighting for this task. SVM was implemented using scikit-learn [69].

ANN: ANN, also known as multi-layer perceptron, produces a mapping from the input space to the target space by optimizing the parameters of the network—the weights and biases connecting nodes in successive layers [70,71]. The ANN architecture was guided by the input size and the number of output classes. The LBFGS solver [72], which is suited for small ANNs trained on datasets, was used for optimizing the log-loss function by adjusting the weights and biases. Rectified Linear Unit (ReLU) activation function was used for all the nodes. The input layer has 210 nodes, followed by two hidden layers with 70 and 4 nodes, respectively, which are followed by the output layer with 2 nodes. ANN was also implemented using the scikit-learn python library [69].

## 3. Experimental Setup

As depicted in Figure 2, the raw EEG data was converted into a series of feature vectors through preprocessing and feature extraction. In the preprocessing stage, the raw EEG data was windowed into 5 s long epochs with no overlap between successive epochs. In the feature extraction stage, *CD* and *SampEn* were calculated on the preprocessed data per epoch for each of the 21 channels. The remaining features were computed from the DWD coefficients. For each session, we had a set of seizure vectors and a set of normal vectors. Since there was an overwhelming amount of normal data, we randomly chose a subset of normal vectors, which was three times the size of the seizure set, for our experiments. Since we have hundreds of hours of data, and 21 channels per epoch, we implemented a map-reduce framework using Python’s Multiprocessing module to make use of multiple processors and speed up the feature extraction by a factor of 10. This framework can be translated into hardware for real-time implementation using an FPGA. For our work, the feature vectors were stored in hdf5 format [73], which optimized memory utilization and execution speed.

We used the NeuroStack hardware for implementing the k-NN and RCE networks. The hardware has the following constraint: each board can commit a maximum of 4096 examples to memory. Hence, a k-NN network on this hardware can store only the first 4096 training vectors. The RCE network stores the first 4096 vectors that do not fall into each other’s influence field. The training order for k-NN was seizure vectors followed by normal vectors. This was done to make sure the k-NN saved sufficient seizure examples in memory. In case of RCE, since the decision space changes with the order of the training vectors, we performed iterative training until two successive iterations resulted in the same decision space.

As mentioned, NeuroStack uses parallel neuromorphic architecture. The basic operation, which is computing the distance between an input example and all the saved examples, takes a constant amount of time, irrespective of the number of saved examples. This is possible because each example is saved in a separate uniquely addressable memory location. This results in a small training time and a quick response while testing. A consequence of this memory setting is that there is a maximum limit on the size of an example. In NeuroStack, each example can be 256 bytes long. To conform to the 256-byte memory limit, every feature was normalized to 255 so that each feature takes up at most 1 byte of memory. This allowed for a maximum of 12 features per channel, which is equivalent to a maximum of 252 bytes per epoch.

## 4. Experiments

The following experiments were performed:

- Feature selection: Filter and wrapper feature selection methods were used to select optimal feature sets. The performance of classifiers was compared for all feature sets;
- Resolution strategy: Different resolution strategies were compared for examples classified as “Unknown” by RCE;
- Number of nearest neighbors: k-NN and RCE networks with varying number of nearest neighbors were compared;
- Number of EEG sessions used for training: Performance of different classifiers was compared for different training-set sizes;
- Varying epoch duration: Performance of a classifier was tracked as the epoch duration was changed;
- Individual vs. population-based systems: Performance of a general classifier trained with data from all individuals was compared to specific classifiers trained with data from each individual.

Training and test set used in the experiments For all the individual-based classifiers, $N_{i-1}$ sessions were used for training and the one remaining session was used for testing, where $N_{i}$ is the number of EEG sessions for individual *i*. For experiments with varying number of sessions used for training, $N_{i}-m$ sessions were used for training and the remaining m sessions were used for testing, for $m\in [1,2,\ldots ,N_{i-1}]$. The experiment with a certain value of m was repeated until every session of an individual was tested at least once.

For population-based classifiers, the training set included session data from all the individuals but one, and all the sessions belonging to the one individual were used for testing. This experiment was repeated for all individuals.

In all the classification experiments, seizure and normal data were respectively designated as the positive and negative classes. The notion of True Positives (*TP*), False Negatives (*FN*), True Negatives (*TN*), and False Positives (*FP*) pertaining to a classifier were defined as:

- *TP*: Number of seizure examples classified as seizure;
- *FN*: Number of seizure examples classified as normal;
- *TN*: Number of normal examples classified as normal;
- *FP*: Number of normal examples classified as seizure.

The following are the metrics used for evaluating and comparing the performance of the classifiers:

*Sensitivity*: Also known as the true positive rate, is the fraction of the seizure examples classified as a seizure.

(7) $$Sensitivity=\frac{TP}{TP+TN}\times 100\%,$$

*Specificity*: Also known as the true negative rate, it is the fraction of normal examples classified as normal.

(8) $$Specificity=\frac{TN}{TN+FP}\times 100\%,$$

*F1-score*: This combines the sensitivity and specificity into a single metric, making it easy to compare the performance of classifiers with different sensitivity and specificity values.

(9) $$F1-score=2\times \frac{Precision\times Recall}{Precision+Recall}\times 100\%,$$

where the *precision* is given by:

(10) $$Precision=\frac{TP}{TP+FP}\times 100\%,$$

and the *recall* is given by:

(11) $$Recall=\frac{TP}{TP+FN}\times 100\%,$$

## 5. Results

### 5.1. Feature Selection

**Figure 6:**
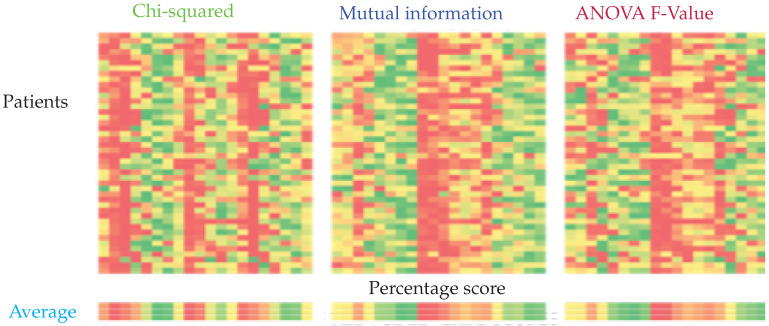
Feature importance calculated using chi-squared, mutual information, and ANOVA F-Value from left to right. The columns represent the relative importance of features for each patient. The importance is represented on a scale from green to red, green being the most important and red the least important.

Each filter-based feature selection system outputs a feature importance list for each individual. It was observed that the optimal feature set differs among individuals, as can be seen in Figure 6. We averaged the importance scores over all individuals for each method and compared the performance of the classifiers with these feature sets.

It can be seen that the top 10 features selected by ANOVA-F and mutual information-based methods are the same, while the Chi-squared based method yields a different set of features. Recursive feature elimination (RFE) was performed for SVM, k-NN, and RCE. It was not performed for ANN since the architecture of ANN would have to change for every different input size. For RCE, the examples classified as Unknown were assigned to normal class. For both RCE and k-NN, one nearest neighbor was used. Table 3 lists the feature sets obtained from the feature selection experiments.

### 5.2. Resolution Strategy

As mentioned in the machine learning section, the examples classified as Unknown by RCE can be resolved via various strategies. The results of using three different strategies with individual-based RCE and RCE RFE feature sets are shown in Table 4. Note that the results are presented for the entire data. Each individual-based RCE results in a pair of sensitivity and specificity values (one for each patient). The variance in the sensitivity and specificity metrics shown in the table is the statistical variance of sensitivity and specificity values across all the patients. It has been observed that using a population-based k-NN on examples classified as Unknown by individual-based RCE results in the best classifier system. One nearest neighbor is used in this case.

### 5.3. Number of Nearest Neighbors

Table 5 shows the performance of individual-based k-NN and RCE networks with a different number of nearest neighbors. We extended the strategy of using a general classifier on Unknown examples in case of RCE to SVM and ANN. For this task, we set a threshold on the predict probability of the class output by SVM/ANN. We observed that most of the non-seizure examples which were classified as seizure had a predicted probability of <0.8. Since the tracer injection is most effective when administered at the actual onset of seizure, we would want a high specificity. To rectify such misclassifications, we decided to input all the examples with predict-probability of <0.8 to a subsequent (population-based) classifier—this resulted in a lower sensitivity but a higher specificity. The improvement observed using this strategy is shown in Table 6, and all the experiments with SVM and ANN presented here use this two-stage approach.

Once the architectures of all the classifiers were set, we compared the performance of the classifiers with different feature sets for individual-based scenario, as presented in Table 7. Following this experiment, the optimal feature set for each classifier was selected. The rest of the experiments were performed in an individual-based scenario using these optimal features, unless otherwise mentioned.

**Figure 7:**
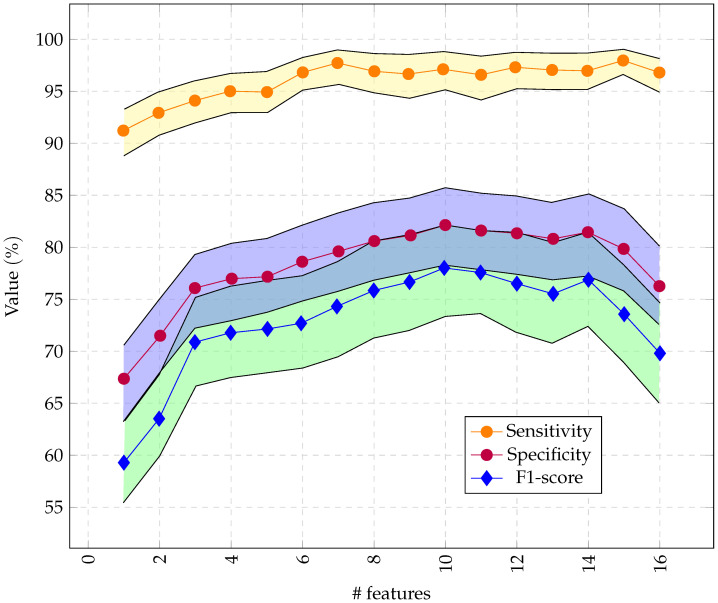
Performance of the RCE system with incremental feature vector sizes, starting with the most important features. Only 16 channels were used so the feature vector did not exceed 256 bytes.

As illustrated in Figure 7, we had optimal performance for RCE with 10 features obtained through RFE.

### 5.4. Number of EEG Sessions Used for Training

The effect of a small amount of training data on the classifier performance is shown in Table 8. For training with multiple sessions, say m out of a total of *N* sessions available per individual, we performed ${(}_{m}^{N})C$ experiments, with every combination of m sessions used for training and the rest used for testing, and averaged the results.

The results for epoch length selection can be found in Table 9. It can be seen also that the specificity remains almost the same as we increase the epoch duration from 1 to 7 s. For sensitivity, as we increased the epoch duration from 1 s to 5 s, the sensitivity increased to reach its maximum at 5 s. For an epoch duration of 6 s and 7 s, the sensitivity decreased. In short, we obtained the best performance with 5 s epoch duration (91.14% and 98.77%).

### 5.5. Individual vs. Population-Based Systems

Table 10 summarizes the final experimental results, in terms of sensitivity and specificity. As can be seen from the table, the RCE network trained on each individual has the best performance with a sensitivity of 80.16% and a specificity of 97.17%, followed by SVM. SVM has the best performance among population-based methods, but it is far from optimal. RCN still has the highest specificity (86.70%) but its sensitivity drops to 42.89%. ANN has the highest specificity for population based method with 81.42%.

### 5.6. Latency for NeuroStack

For epoch duration of 5 s, the feature extraction process, when parallelized with four threads, took 11.6±0.1 s. The majority of the time (∼8 s) was taken to calculate the Correlation Dimension. If *CD* was not calculated, the latency for 5 s epoch duration was 3.6±0.2 s. Exclusion of *CD* from the feature set resulted in a 3% drop in sensitivity and 1% drop in specificity. The classification process took 1.2 μs on an average. The latency can be reduced with smaller epoch duration, but with a small drop in sensitivity. Latency has a dependency on epoch size. For example, using 2 s epoch duration yields a latency of ∼5 s (including computation of *CD*).

## 6. Discussion

The experiments conducted in this work and the results provide relevant information for deciding the architecture of the classifier and the overall real-time seizure detection system. It can be observed that RCE has better performance than SVM, ANN, and k-NN when trained with data from a single session, and this performance is comparable to the one obtained using all sessions for training. In the case of SVM and ANN, we see that the performance gradually improves with data size, and they need numerous EEG sessions for training to reach the performance obtained by RCE with a training set composed of a single EEG session. This performance disparity can be attributed to the ability of RCE networks to identify anomalies with confidence, even with a small amount of seizure data. Having a secondary population-based classifier to classify the Unknown examples (or examples with predict probability <0.8 in case of ANN and SVM) also improved the performance of the classification systems. All the examples which could not be classified by the primary model with a high probability were input to a secondary classifier for a second opinion and classified accordingly. It can be seen from Table 10 that individual-based seizure detection systems work better than population-based systems. This can be attributed to the high variability in the seizure patterns from person to person, which cannot be captured by a single general model. In this study, the seizure and normal data was labeled and we knew the start and end of each type of data. Hence, non-overlapping epochs were used without any performance degradation. When deployed in a clinical workflow, the system will be working with continuous streams of EEG data. Overlapping epochs can be used in such a scenario to further reduce the chance of missing a seizure since there will be more seizure epochs. The present system can be further improved by pre-processing the data to remove artifacts using combined Blind source separation and independent component analysis [74], which has proven more useful for separating linearly mixed independent sources in EEG signals, including artifacts [75,76]. The influence of such pre-processing can improve the presented results and can be investigated in future studies.

This improved system can be used to trigger tracer injection for ictal-SPECT. In the future, our system can also be used to trigger deep brain stimulation (electro-stimulation) for suppressing ictal discharges and their propagation, and to inject drugs intracranially for more effective seizure control.

Table 11 presents a summary of the main results presented in this study in comparison between the proposed techniques and the state of the art techniques. We did not implement the techniques using the hardware as some of the techniques are not applicable to our study. However, we cite the results obtained by several papers recently published for the sake of the comparison. It can be seen from Table 11, that the proposed method is able to achieve over 90% plus sensitivity, specificity, and accuracy, all while keeping the delay of the seizure detection within 12 s.

## 7. Conclusions

This study presents an approach for automatic seizure detection based on RCN networks. These networks are data efficient. This study is the first of its kind in terms of hardware implementation and validation of the theoretical approach. The proposed methods are comparable to recent deep learning techniques that can achieve state of the art detection accuracy. The proposed technique has the advantage of being trained on fewer training samples instead of large database required by deep learning, which entails tedious labeling work. It can be concluded that a 5 s epoch duration resulted in the highest sensitivity and specificity. It can be concluded that the latency of RCE is highly dependent on the *CD* feature. Including the *CD* feature improves the system accuracy but at the cost of increased latency. Also, individual-based RCN has better performance than population-based RCE. It can be concluded also that increasing the number of EEG sessions resulted in better performance for all the studied algorithms. Increasing the number of neighbors results in an increase of specificity of K-NN but not the sensitivity.

## Figures and Tables

**Table 1:** State-of-the-art seizure detection techniques.

Seizure Detection Approach	Model	Structure	Metrics	Comments
	Support Vector Machine [11,35]	Selective Search Algorithm + SVM/ Genetic algorithm + SVM	Achieves an accuracy of 99%	Method explored double-density discrete wavelet transform (DD-DWT); designing GA objective function can be difficult
	Random Forest [20]	Random Forest + grid search	Achieves true probability of serious epilepsy 98%	Grid search takes a large amount of training time to traverse all the grid parameters
Machine Learning	K-Neighrest Neighbor and Genetic Algorithms [31,36]	Hilbert transform	The average accuracy of our proposed scheme is as high as 91.33%	No latency reported
	Neural Networks [14]	multilayer perceptron neural network with single hidden layer of 10 neurons	Yields sensitivity, specificity, and a false detection rate of 97.1%, 97.8%, and 1 h${}^{-1}$, respectively	The method is not fully automated
	ANN+SVM [37]	5-level Db4 discrete wavelet transform, GA-based feature selection and ANN/SVM classifiers	Achieves	No latency reported
Unsupervised ML	XGBoost [38]	4597 EEG files train, 1013 EEGs validation, and 1026 EEG files test	XGBoost-based method achieved sensitivity and false alarm/24 h of 20.00% and 15.59, respectively, in the test set	XGBoost has the potential of improvement, but requires adding more training data
	2D-CNN [24]	2D deep CNN architecture	Specificity of 90%	
	VGGNet [25]	Lightweight VGGNet trained on Global MIC	Achieves a sensitivity and Specificity of 90%	Require a sizable dataset
Deep Learning	Deep Learning +LSTM [23]	CNN	Achieves sensitivity and specificity of 98%	
	CNN [26]	CNN	Achieves sensitivity and specificity of 64.96%	Accuracies of time domain signals are significantly decreased compared to frequency domain signals
Wavelet-Based Method	Wavelet-based [39]	Wavelet Transform + SVM	Achieves a sensitivity of 94.46% and a specificity of 95.26% with a false detection rate of 0.58/h	None
	Wavelet + SVM [40]	Wavelet + SVM	A specificity of 100%, sensitivity of 97.2% and an accuracy of 98.6% were obtained.	None
Non-ML methods	ICON Method [41]	Inferring connection networks used with CHB-MIT scalp EEG database (https://physionet.org/content/chbmit/1.0.0/, accessed on 22 February 2022)	Sensitivity of 93.6% and false positive rate of 0.16 per hour	Method is hard to generalize

**Table 2:** Coefficients of signals obtained from six levels of discrete wavelet decomposition and the frequency range represented by each set of coefficients.

Coefficients	Frequency Range (Hz)
1st level detail (D1)	[125, 250]
2nd level detail (D2)	[62.5, 125]
3rd level detail (D3)	[31.25, 62.5]
4th level detail (D4)	[15.625, 31.25]
5th level detail (D5)	[7.8125, 15.625]
6th level detail (D6)	[3.90625, 7.8125]
6th level approximation (A6)	[0, 3.90625]

**Table 3:** Composition of different feature sets.

Feature Set	Composition
ANOVA-F	*CD*, SampEN, RMS (D4, D3, D2, D1)
Chi-squared	RMS (D4, D3, D2), MA (D4, D3, D2, D1)
SVM RFE	*SampEn*, RMS (D4, D2, D1), MA (D1), AM (D2, D1)
k-NN RFE	*CD*, *SampEn*, RMS (D6, D5, D4, D3, D2, D1)
RCE RFE	*CD*, *SampEn*, RMS (D2, D1), AM (D6, D5, D4, D3, D2, D1)

**Table 4:** Performance of RCE network with different “Unknown” resolution strategies.

Resolution Strategy	Sensitivity (%)	Specificity (%)
Assign to Seizure class	88.59 ± 7.23	69.18 ± 15.78
Assign to Normal class	66.23 ± 17.09	91.51 ± 9.47
Use population k-NN	80.16 ± 4.50	97.17 ± 1.02

**Table 5:** Performance (sensitivity/specificity) of k-NN and RCE networks for a different number of nearest neighbors (*k*).

# Nearest Neighbors (*k*)	k-NN	RCE
1	57.56/84.65	80.16/97.17
2	57.56/84.65	80.16/97.17
3	45.46/91.06	77.91/97.20
4	47.60/90.95	80.44/97.43
5	37.86/92.13	80.15/96.99
6	40.12/91.81	81.61/97.22
7	31.85/94.58	79.59/97.30

**Table 6:** Comparison of single and two-stage SVM and ANN classifiers with the SVM RFE feature set.

Type	Sensitivity (%)	Specificity (%)
Single-stage SVM	90.90 ± 8.09	78.03 ± 12.82
Two-stage SVM	80.17 ± 10.91	93.03 ± 2.91
Single-stage ANN	60.60 ± 20.78	85.90 ± 18.08
Two-stage ANN	88.62 ± 10.90	81.41 ± 16.78

**Table 7:** Comparison of single and two-stage SVM and ANN classifiers with SVM RFE feature set (metric format: Sensitivity (%)/Specificity (%)).

Feature Set	SVM	ANN	k-NN	RCE
ANOVA	78.56/93.28	90.97/78.90	54.81/72.27	74.32/98.38
Chi-sq	85.68/81.64	90.18/80.75	48.15/77.74	76.14/95.16
SVM RFE	80.17/93.03	88.62/81.47	54.05/73.79	75.47/96.03
k-NN RFE	85.44/81.93	76.77/82.20	57.56/84.65	74.20/88.32
RCE RFE	87.94/82.36	85.22/75.51	55.47/70.36	80.16/97.17

**Table 8:** Performance (sensitivity/specificity rounded to the nearest integer) of different classifiers with varying number of EEG sessions used for training.

# Training Sessions	SVM	ANN	k-NN	RCE
1	52/93	45/79	55/71	75/89
2	70/88	72/77	53/80	77/93
N−1	80/93	89/81	58/85	80/97

**Table 9:** Performance of individual-based RCE classifier system (over a subset of individuals) for different epoch durations.

Epoch Duration (s)	Sensitivity (%)	Specificity (%)
1	79.78 ± 1.05	97.16 ± 0.30
2	80.44 ± 1.34	97.32 ± 0.38
3	81.32 ± 1.83	97.25 ± 0.47
4	83.11 ± 1.97	97.62 ± 0.57
5	91.14 ± 1.77	98.77 ± 0.57
6	87.34 ± 2.61	98.08 ± 0.64
7	86.25 ± 1.90	97.33 ± 0.81

**Table 10:** Optimal performance of different classifiers with individual- and population-based training.

System	Sensitivity (%)	Specificity (%)
Individual-based RCE	80.16 ± 4.50	97.17 ± 1.02
Individual-based k-NN	57.56 ± 16.46	84.65 ± 14.60
Individual-based ANN	88.62 ± 10.90	81.41 ± 16.78
Individual-based SVM	80.17 ± 10.91	93.03 ± 2.91
Population-based RCE	42.89 ± 38.80	86.70 ± 12.55
Population-based k-NN	61.73 ± 23.09	67.44 ± 23.52
Population-based ANN	81.42 ± 6.69	32.57 ± 19.49
Population-based SVM	66.27 ± 32.44	72.70 ± 22.18

**Table 11:** Performance comparison between the state of the art techniques and proposed methods.

Methods	Approach	Sensitivity (%)	Specificity(%)	Accuracy (%)	Delay (s)
[29]	ML	NA	NA	93.82	N/A
[23]	DL + LSTM	98.72	98.86	98.79	N/A
[24]	2D CNN	90.0	91.05	98.05	N/A
Proposed Method 1	RCE with multi-features	91.14	98.77	-	11 s
Proposed Method 2	RCE-without *CD*	80.116	97.17	-	3.6 s

## Abbreviations

The following abbreviations are used in this manuscript:

RCE	Reduced Columb Energy
k-NN	k-Nearest Neighbors
SVM	Support Vector Machines
ANN	Artificial Neural Networks
RFE	Recursive Feature Elimination
EEG	Electroencephalogram
DWD	Discrete Wavelet Decomposition
*CD*	Correlation Dimension
FPGA	Field Programmable Gate Arrays
NN	Neural Network
*TP*	True Positive
*FN*	False Negative
*TN*	True Negative
*FP*	False Positive
ictal-SPECT	ictal Single Photon Emission Computed Tomography
CNN	Convolutional Neural Network
LSTM	Long short-term memory
RMS	Root mean square
MA	Mean of the absolute values
AM	Absolute value of the mean
GUI	Graphical user interface
Hz	Hertz
*SampEn*	Sample entropy
IF	Influence field
DL	Deep Learning
ML	Machine Learning

## References

[1] Aarabi A., Fazel-Rezai R., Aghakhani Y. (2009). A fuzzy rule-based system for epileptic seizure detection in intracranial EEG. Clin. Neurophysiol..

[2] Kwan P., Brodie M.J. (2000). Early identification of refractory epilepsy. N. Engl. J. Med..

[3] Duncan J. (2009). The current status of neuroimaging for epilepsy. Curr. Opin. Neurol..

[4] Roy C.S., Sherrington C.S. (1890). On the regulation of the blood-supply of the brain. J. Physiol..

[5] Oommen K.J., Saba S., Oommen J.A., Francel P.C., Arnold C.D., Wilson D.A. (2004). The relative localizing value of interictal and immediate postictal SPECT in seizures of temporal lobe origin. J. Nucl. Med..

[6] Pastor J., Domínguez-Gadea L., Sola R.G., Hernando V., Meilán M.L., De Dios E., Martínez-Chacón J.L., Martínez M. (2008). First true initial ictal SPECT in partial epilepsy verified by electroencephalography. Neuropsychiatr. Dis. Treat..

[7] Setoain X., Pavía J., Serés E., Garcia R., Carreño M.M., Donaire A., Rubí S., Bargalló N., Rumià J., Boget T. (2012). Validation of an automatic dose injection system for Ictal SPECT in epilepsy. J. Nucl. Med..

[8] Ho S.S., Berkovic S.F., McKay W.J., Kalnins R.M., Bladin P.F. (1996). Temporal lobe epilepsy subtypes: Differential patterns of cerebral perfusion on ictal SPECT. Epilepsia.

[9] Orosco L., Correa A.G., Laciar E. (2013). A survey of performance and techniques for automatic epilepsy detection. J. Med. Biol. Eng..

[10] Subasi A. (2007). EEG signal classification using wavelet feature extraction and a mixture of expert model. Expert Syst. Appl..

[11] Fu K., Qu J., Chai Y., Zou T. (2015). Hilbert marginal spectrum analysis for automatic seizure detection in EEG signals. Biomed. Signal Process. Control.

[12] Elgammal M.A., Mostafa H., Salama K.N., Nader Mohieldin A. A Comparison of Artificial Neural Network(ANN) and Support Vector Machine(SVM) Classifiers for Neural Seizure Detection. Proceedings of the 2019 IEEE 62nd International Midwest Symposium on Circuits and Systems (MWSCAS).

[13] Shankar R.S., Raminaidu C., Raju V.S., Rajanikanth J. (2021). Detection of Epilepsy based on EEG Signals using PCA with ANN Model. J. Phys..

[14] Sriraam N., Raghu S., Tamanna K., Narayan L., Khanum M., Hegde A., Kumar A.B. (2018). Automated epileptic seizures detection using multi-features and multilayer perceptron neural network. Brain Inform..

[15] Shoeb A., Edwards H., Connolly J., Bourgeois B., Treves S.T., Guttag J. (2004). Patient-specific seizure onset detection. Epilepsy Behav..

[16] Qu H., Gotman J. (1997). A patient-specific algorithm for the detection of seizure onset in long-term EEG monitoring: Possible use as a warning device. IEEE Trans. Biomed. Eng..

[17] Georgiy R., John B., Martha J., Richard N. (2010). Patient-specific early seizure detection from scalp electroencephalogram. J. Clin. Neurophysiol..

[18] Kiranyaz S., Ince T., Zabihi M., Ince D. (2014). Automated patient-specific classification of long-term electroencephalography. J. Biomed. Inform..

[19] Baumgartner C., Koren J.P. (2018). Seizure detection using scalp-EEG. Epilepsia.

[20] Wang X., Gong G., Li N., Qiu S. (2019). Detection analysis of epileptic EEG using a novel random forest model combined with grid search optimization. Front. Hum. Neurosci..

[21] Fergus P., Hussain A., Hignett D., Al-Jumeily D., Abdel-Aziz K., Hamdan H. (2016). A machine learning system for automated whole-brain seizure detection. Appl. Comput. Inform..

[22] Lippmann R. (1989). Pattern classification using neural networks. IEEE Commun. Mag..

[23] Abdelhameed A., Bayoumi M. (2021). A deep learning approach for automatic seizure detection in children with epilepsy. Front. Comput. Neurosci..

[24] Hossain M.S., Amin S.U., Alsulaiman M., Muhammad G. (2019). Applying deep learning for epilepsy seizure detection and brain mapping visualization. ACM Trans. Multimed. Comput. Commun. Appl..

[25] Ke H., Chen D., Li X., Tang Y., Shah T., Ranjan R. (2018). Towards brain big data classification: Epileptic EEG identification with a lightweight VGGNet on global MIC. IEEE Access.

[26] Zhou M., Tian C., Cao R., Wang B., Niu Y., Hu T., Guo H., Xiang J. (2018). Epileptic seizure detection based on EEG signals and CNN. Front. Neuroinform..

[27] Liao Y. (2001). Neural Networks in Hardware: A Survey.

[28] Duda R.O., Hart P.E., Stork D.G. (2012). Pattern Classification.

[29] Wang L., Xue W., Li Y., Luo M., Huang J., Cui W., Huang C. (2017). Automatic epileptic seizure detection in EEG signals using multi-domain feature extraction and nonlinear analysis. Entropy.

[30] Shoka A.A.E., Alkinani M.H., El-Sherbeny A., El-Sayed A., Dessouky M.M. (2021). Automated seizure diagnosis system based on feature extraction and channel selection using EEG signals. Brain Inform..

[31] Rivero D., Fernandez-Blanco E., Dorado J., Pazos A. A new signal classification technique by means of Genetic Algorithms and kNN. Proceedings of the 2011 IEEE Congress of Evolutionary Computation (CEC).

[32] Morabito F.C., Andreou A.G., Chicca E. (2013). Neuromorphic engineering: From neural systems to brain-like engineered systems. Neural Netw..

[33] Sharifshazileh M., Burelo K., Sarnthein J., Indiveri G. (2021). An electronic neuromorphic system for real-time detection of high frequency oscillations (HFO) in intracranial EEG. Nat. Commun..

[34] General Vision, Inc NeuroStack Hardware Manual. https://www.general-vision.com/documentation/TM_NeuroStack_Hardware_Manual.pdf.

[35] Li M., Chen W., Zhang T. (2016). Automatic epilepsy detection using wavelet-based nonlinear analysis and optimized SVM. Biocybern. Biomed. Eng..

[36] Vipani R., Hore S., Basak S., Dutta S. Detection of epilepsy using Hilbert transform and KNN based classifier. Proceedings of the 2017 IEEE International Conference on Smart Technologies and Management for Computing, Communication, Controls, Energy and Materials (ICSTM).

[37] Omidvar M., Zahedi A., Bakhshi H. (2021). EEG signal processing for epilepsy seizure detection using 5-level Db4 discrete wavelet transform, GA-based feature selection and ANN/SVM classifiers. J. Ambient. Intell. Humaniz. Comput..

[38] Wei L., Mooney C. Epileptic Seizure Detection in Clinical EEGs Using an XGboost-based Method. Proceedings of the 2020 IEEE Signal Processing in Medicine and Biology Symposium (SPMB).

[39] Liu Y., Zhou W., Yuan Q., Chen S. (2012). Automatic seizure detection using wavelet transform and SVM in long-term intracranial EEG. IEEE Trans. Neural Syst. Rehabil. Eng..

[40] Fathima T., Rahna P., Gaffoor T. Wavelet based detection of epileptic seizures using scalp EEG. Proceedings of the 2020 International Conference on Futuristic Technologies in Control Systems Renewable Energy (ICFCR).

[41] Bomela W., Wang S., Chou C.A., Li J.S. (2020). Real-time inference and detection of disruptive EEG networks for epileptic seizures. Sci. Rep..

[42] Jasper H.H. (1958). The ten-twenty electrode system of the International Federation. Electroencephalogr. Clin. Neurophysiol..

[43] Krishnaveni V., Jayaraman S., Aravind S., Hariharasudhan V., Ramadoss K. (2006). Automatic identification and removal of ocular artifacts from EEG using wavelet transform. Meas. Sci. Rev..

[44] Jiang X., Bian G.B., Tian Z. (2019). Removal of artifacts from EEG signals: A review. Sensors.

[45] Rafiee J., Rafiee M., Prause N., Schoen M. (2011). Wavelet basis functions in biomedical signal processing. Expert Syst. Appl..

[46] Percival D.B., Walden A.T. (2000). Wavelet Methods for Time Series Analysis.

[47] Greco A., Costantino D., Morabito F., Versaci M. A Morlet wavelet classification technique for ICA filtered SEMG experimental data. Proceedings of the International Joint Conference on Neural Networks.

[48] Aarabi A., He B. (2012). A rule-based seizure prediction method for focal neocortical epilepsy. Clin. Neurophysiol..

[49] Adeli H., Ghosh-Dastidar S., Dadmehr N. (2007). A wavelet-chaos methodology for analysis of EEGs and EEG subbands to detect seizure and epilepsy. IEEE Trans. Biomed. Eng..

[50] Bhavsar R., Helian N., Sun Y., Davey N., Steffert T., Mayor D. (2018). Efficient methods for calculating sample entropy in time series data analysis. Procedia Comput. Sci..

[51] Richman J.S., Moorman J.R. (2000). Physiological time-series analysis using approximate entropy and sample entropy. Am. J. Physiol.-Heart Circ. Physiol..

[52] Song Y., Liò P. (2010). A new approach for epileptic seizure detection: Sample entropy based feature extraction and extreme learning machine. J. Biomed. Sci. Eng..

[53] Grassberger P., Procaccia I. (2004). Measuring the strangeness of strange attractors. The Theory of Chaotic Attractors.

[54] Brari Z., Belghith S. (2021). A novel Machine Learning approach for epilepsy diagnosis using EEG signals based on Correlation Dimension. IFAC-PapersOnLine.

[55] Zandi A.S., Javidan M., Dumont G.A., Tafreshi R. (2010). Automated real-time epileptic seizure detection in scalp EEG recordings using an algorithm based on wavelet packet transform. IEEE Trans. Biomed. Eng..

[56] Faust O., Acharya U.R., Adeli H., Adeli A. (2015). Wavelet-based EEG processing for computer-aided seizure detection and epilepsy diagnosis. Seizure.

[57] Chen D., Wan S., Xiang J., Bao F.S. (2017). A high-performance seizure detection algorithm based on Discrete Wavelet Transform (DWT) and EEG. PLoS ONE.

[58] Dutta T. Dynamic Time Warping Based Approach to Text-Dependent Speaker Identification Using Spectrograms. Proceedings of the 2008 Congress on Image and Signal Processing.

[59] Magosso E., Ursino M., Zaniboni A., Gardella E. (2009). A wavelet-based energetic approach for the analysis of biomedical signals: Application to the electroencephalogram and electro-oculogram. Appl. Math. Comput..

[60] Shoeb A.H., Guttag J.V. Application of machine learning to epileptic seizure detection. Proceedings of the ICML’10: 27th International Conference on International Conference on Machine Learning.

[61] Kraskov A., Stögbauer H., Grassberger P. (2004). Estimating mutual information. Phys. Rev. E.

[62] Ross B.C. (2014). Mutual information between discrete and continuous data sets. PLoS ONE.

[63] Cochran W.G. (1952). The *χ*2 test of goodness of fit. Ann. Math. Stat..

[64] Heiman G.W. (2001). Understanding Research Methods and Statistics: An Integrated Introduction for Psychology.

[65] Lowry R. Concepts and Applications of Inferential Statistics. http://vassarstats.net/textbook/.

[66] Guyon I., Weston J., Barnhill S., Vapnik V. (2002). Gene selection for cancer classification using support vector machines. Mach. Learn..

[67] Dinstein I., Heeger D.J., Behrmann M. (2015). Neural variability: Friend or foe?. Trends Cogn. Sci..

[68] Cortes C., Vapnik V. (1995). Support-vector networks. Mach. Learn..

[69] Pedregosa F., Varoquaux G., Gramfort A., Michel V., Thirion B., Grisel O., Blondel M., Prettenhofer P., Weiss R., Dubourg V. (2011). Scikit-learn: Machine learning in Python. J. Mach. Learn. Res..

[70] Dreyfus S.E. (1990). Artificial neural networks, back propagation, and the Kelley-Bryson gradient procedure. J. Guid. Control. Dyn..

[71] Hinton G.E. (1990). Connectionist learning procedures. Machine Learning.

[72] Mokhtari A., Ribeiro A. (2015). Global convergence of online limited memory BFGS. J. Mach. Learn. Res..

[73] Group H. Hierarchical Data Format, Version 5 (1997–2017). https://asdc.larc.nasa.gov/documents/tools/hdf.pdf.

[74] Comon P. (1994). Independent component analysis, a new concept?. Signal Process..

[75] Barborica A., Mindruta I., Sheybani L., Spinelli L., Oane I., Pistol C., Donos C., López-Madrona V.J., Vulliemoz S., Bénar C.G. (2021). Extracting seizure onset from surface EEG with Independent Component Analysis: Insights from simultaneous scalp and intracerebral EEG. NeuroImage Clin..

[76] Jung T.P., Makeig S., Humphries C., Lee T.W., Mckeown M.J., Iragui V., Sejnowski T.J. (2000). Removing electroencephalographic artifacts by blind source separation. Psychophysiology.

